# Supplementary material for: Causal Relationship between Aging and Anorexia Nervosa: A White-Matter-Microstructure-Mediated Mendelian Randomization Analysis
Source: Biomedicines. 2024 Aug 16;12(8):1874. doi: 10.3390/biomedicines12081874 (PMC11351342; doi:10.3390/biomedicines12081874)
Supplement: Supplementary file 1 [file biomedicines-12-01874-s001.zip › Supplementary Figure/Figure S4/Singe SNP plot of MR analysis for TL to ubm-b-1711.pdf]

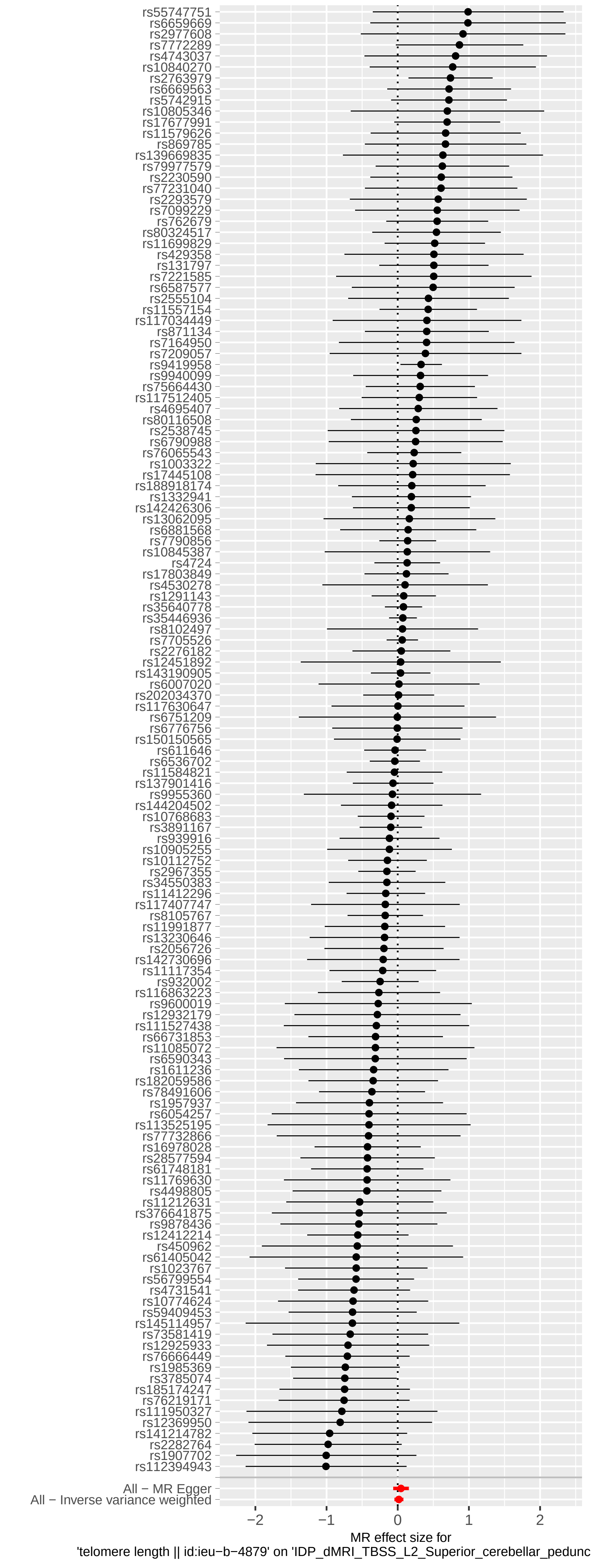
rs55747751
rs6659669
rs2977608
rs7772289
rs4743037
rs10840270
rs2763979
rs6669563
rs5742915
rs10805346
rs17677991
rs11579626
rs869785
rs139669835
rs79977579
rs2230590
rs77231040
rs2293579
rs7099229
rs762679
rs80324517
rs11699829
rs429358
rs131797
rs7221585
rs6587577
rs2555104
rs11557154
rs117034449
rs871134
rs7164950
rs7209057
rs9419958
rs9940099
rs75664430
rs117512405
rs4695407
rs80116508
rs2538745
rs6790988
rs76065543
rs1003322
rs17445108
rs188918174
rs1332941
rs142426306
rs13062095
rs6881568
rs7790856
rs10845387
rs4724
rs17803849
rs4530278
rs1291143
rs35640778
rs35446936
rs8102497
rs7705526
rs2276182
rs12451892
rs143190905
rs6007020
rs202034370
rs117630647
rs6751209
rs6776756
rs150150565
rs611646
rs6536702
rs11584821
rs137901416
rs9955360
rs144204502
rs10768683
rs3891167
rs939916
rs10905255
rs10112752
rs2967355
rs34550383
rs11412296
rs117407747
rs8105767
rs11991877
rs13230646
rs2056726
rs142730696
rs11117354
rs932002
rs116863223
rs9600019
rs12932179
rs111527438
rs66731853
rs11085072
rs6590343
rs1611236
rs182059586
rs78491606
rs1957937
rs6054257
rs113525195
rs77732866
rs16978028
rs28577594
rs61748181
rs11769630
rs4498805
rs11212631
rs376641875
rs9878436
rs12412214
rs450962
rs61405042
rs1023767
rs56799554
rs4731541
rs10774624
rs59409453
rs145114957
rs73581419
rs12925933
rs76666449
rs1985369
rs3785074
rs185174247
rs76219171
rs111950327
rs12369950
rs141214782
rs2282764
rs1907702
rs112394943
All − MR Egger
All − Inverse variance weighted
−2
−1
0
1
2
MR effect size for
'telomere length || id:ieu−b−4879' on 'IDP_dMRI_TBSS_L2_Superior_cerebellar_pedunc
